# Choice of anesthetic technique on plasma concentrations of interleukins and cell adhesion molecules

**DOI:** 10.1186/2047-0525-2-8

**Published:** 2013-05-02

**Authors:** Daniela C Ionescu, Simona Claudia D Margarit, Adina Norica I Hadade, Teodora N Mocan, Nicolae A Miron, Daniel I Sessler

**Affiliations:** 1 Department of Anesthesia and Intensive Care I, ‘Iuliu Hatieganu’ University of Medicine and Pharmacy, Croitorilor, nr. 19-21, Cluj-Napoca 400162, Romania; 2 Outcomes Research Consortium, Cleveland, OH, USA; 3 Department of Anaesthesia and Intensive Care, Regional Institute of Gastroenterology and Hepatology‘O Fodor’, Croitorilor, nr. 19-21, Cluj-Napoca 400162, Romania; 4 Department of Physiology, ‘Iuliu Hatieganu’ University of Medicine and Pharmacy, Croitorilor, nr. 19-21, Cluj-Napoca 400162, Romania; 5 Department of Clinical Immunology, ‘Iuliu Hatieganu’ University of Medicine and Pharmacy, Croitorilor, nr. 19-21, Cluj-Napoca 400162, Romania; 6 Department of Outcomes Research, The Cleveland Clinic 9500 Euclid Ave -- P77, Cleveland, OH 44195, USA

**Keywords:** Inhalation anesthetics, Intravenous anesthetics, Propofol, Cell adhesion molecules, Interleukins

## Abstract

**Background:**

Whether inflammatory responses to surgery are comparably activated during total intravenous anesthesia (TIVA) and during volatile anesthesia remains unclear. We thus compared the perioperative effects of TIVA and isoflurane anesthesia on plasma concentrations of proinflammatory and anti-inflammatory interleukins and cell adhesion molecules.

**Methods:**

Patients having laparoscopic cholecystectomies were randomly allocated to two groups: 44 were assigned to TIVA and 44 to isoflurane anesthesia. IL-1β, IL-6, IL-8, IL-10, IL-13, and the cellular adhesion molecules intercellular adhesion molecule-1 and vascular cell adhesion molecule-1 were determined preoperatively, before incision, and at 2 and 24 hours postoperatively. Our primary outcomes were area-under-the-curve cytokine and adhesion molecule concentrations over 24 postoperative hours.

**Results:**

The only statistically significant difference in area-under-the-curve concentrations was for IL-6, which was greater in patients given isoflurane:78 (95% confidence interval (CI): 52 to 109) pg/ml versus 33 (22 to 50) pg/ml, *P*= 0.006. Two hours after surgery, IL-6 was significantly greater than baseline in patients assigned to isoflurane: 47 (95% CI: 4 to 216, *P*<0.001) pg/ml versus 18 (95%CI: 4 to 374, *P*<0.001) pg/ml in the TIVA group. In contrast, IL-10 was significantly greater in patients assigned to TIVA: 20 (95% CI: 2 to 140, *P*<0.001) pg/ml versus 12 (95% CI: 3 to 126, *P*<0.001) pg/ml. By 24 hours after surgery, concentrations were generally similar between study groups and similar to baseline values.

**Conclusion:**

The only biomarker whose postoperative area-under-the-curve concentrations differed significantly as a function of anesthetic management was IL-6. Two hours after surgery, IL-6 concentrations were significantly greater in patients given isoflurane than TIVA. However, the differences were modest and seem unlikely to prove clinically important. Further studies are needed.

## Background

Surgery provokes hemodynamic, metabolic, and inflammatory responses; it also provokes a complex immune reaction [1] that includes activation of the interleukin network [2]. For example, surgery and anesthesia provoke an increase in proinflammatory interleukins and adhesion molecules, and a subsequent increase in countervailing anti-inflammatory interleukins [3-5]. The most important proinflammatory interleukin is IL-6, while the most potent anti-inflammatory interleukin is IL-10.

Cell adhesion molecules (CAMs) are members of the immunoglobulin family and are regulated by the inflammatory interleukins [6]. CAMs promote wound healing [7,8] and can also promote tumor progression and metastasis [9,10], either directly or by promoting overproduction of inflammatory interleukins. The mechanisms of action differ for intercellular adhesion molecule (ICAM)-1 and vascular cell adhesion molecule (VCAM)-1. ICAM-1 produces its effects by promoting recruitment and adhesion of leukocytes to the activated endothelium via bonds with integrins, thus promoting migration through endothelial cells [11]; VCAM-1, produced mainly by the endothelial cells after interleukin-mediated stimulation, promotes adhesion of inflammatory cells to the vascular wall and subsequent migration [12]. Reduced plasma ICAM-1 concentration may facilitate tumor cell movement across vessels into surrounding tissues, thus promoting metastasis. Some tumor cells may also use VCAM-1 to adhere to the vascular walls and to migrate [13,14]. CAMs are influenced by the surgery [15] and may influence outcome after oncological surgery [14-17].

The two major approaches to general anesthesia are volatile anesthetics such as isoflurane and intravenous anesthesia such as propofol. General anesthesia may impair immune function directly by affecting immunocompetent cells such as natural killer (NK) cells and macrophages, cytokine responses, and adhesion molecules. Alternatively, anesthesia may indirectly influence the stress response to surgery [1,5,18,19].

There appear to be substantial differences between intravenous and volatile anesthetics in their effects on various immune functions [20-22]. While isoflurane inhibits interferon stimulation of NK cell cytotoxicity and sevoflurane alters the release of cytokines by NK, propofol does not significantly suppress NK cell activity [19]. Propofol alone increased apoptosis and cell adhesion and did not significantly influenced cell migration in breast cancer cells, while propofol conjugates significantly increased apoptosis and decreased migration and adhesion in breast cancer cells [23]. Propofol down-regulates proinflammatory interleukins [24,25]. Although some studies suggest that total intravenous anesthesia (TIVA) suppresses inflammatory responses and promotes release of anti-inflammatory cytokines [18,20,21], the reported results are inconsistent [26]. The few studies that evaluated the effects of anesthesia on cellular adhesion molecules report that both inhalation agents and propofol blunt the increase in ICAM and VCAM expression [27,28]. Whether interleukins and other inflammatory molecules are comparably activated during TIVA and volatile anesthesia thus remains unclear.

Our study was designed to compare the effects of TIVA and volatile anesthesia on plasma concentrations of proinflammatory and anti-inflammatory interleukins and on CAMs during laparoscopic surgery. Specifically, we tested the hypothesis that TIVA blunts the inflammatory response to laparoscopic cholecystectomy more than isoflurane anesthesia. Laparoscopic surgery was chosen because the amount of tissue injury is small and similar from case to case, thus reducing the impact of surgery *per se* on immune responses.

## Methods

After obtaining university Ethics Committee approval (Comisia de Etica a Universitatii de Medicina si Famacie ‘Iuliu Hatieganu’, Cluj-Napoca, No. 178A/2007) and written informed consent, we enrolled 88 patients with American Society of Anesthesiologists physical status scores 1 or 2 who were scheduled for laparoscopic cholecystectomy. We excluded patients with known inflammatory diseases (including acute cholecystitis) or immune system disorders, asthma, obesity (body mass index ≥30 kg/m^2^), diabetes, gastric ulcers, and allergies. We also excluded patients who currently or recently used steroid or anti-inflammatory medication, or who had white blood cell counts >10^4^/μl or a preoperative core temperature >37°C.

### Protocol

Midazolam, 7.5 mg orally, was given 1 hour before surgery. Dexamethasone, 4 mg intravenously, was given shortly before induction of anesthesia as prophylaxis against postoperative nausea and vomiting [29]. Then 500 ml crystalloid was infused during anesthetic induction, and thereafter as clinically indicated.

Patients were randomized into two study groups using a computer-generated sequence: one-half were assigned to target-controlled infusion TIVA with propofol, and one-half to isoflurane volatile anesthesia. Allocation was concealed in sequentially numbered sealed envelopes, with assignments revealed when patients were arriving in the operating theater. Laboratory staff were blinded to group assignments and anesthetic management.

TIVA was induced and maintained with a target-controlled infusion of propofol with an initial target plasma concentration of 4 μg/ml (Orchestra, Base Primea; Fresenius Kabi, Fresenius Vial SAS, Brézins, France). The propofol infusion was adjusted to target a Bispectral Index between 40 and 55 (Covidien, Dublin, Ireland). Isoflurane anesthesia was induced with propofol 1.5 to 2 mg/kg and maintained with isoflurane 1 to 1.5 minimum alveolar concentration titrated to Bispectral Index 40 to 55 and hemodynamic parameters.

Patients were ventilated with 70% oxygen in air and a positive end-expiratory pressure of 3 to 4 cmH_2_O. The respiratory rate and inspiratory pressure were adjusted to maintain an end-tidal carbon dioxide partial pressure of 35 to 45 mmHg (Drager-Vamos, Lubeck, Germany). Remifentanil was infused under manual control with an initial dose of 0.5 μg/kg/minute in the first minute and 0.25 μg/kg/minute thereafter; the remifentanil dose was adjusted in 0.05 to μg/kg/minute increments as clinically necessary. Atracurium 0.6 mg/kg was initially given to provide paralysis for intubation; subsequently, 10 mg boluses were given as clinically needed. Hypotension (defined as a decrease in blood pressure >30% from baseline) was corrected with additional fluids and/or ephedrine. At the end of surgery, anesthesia was discontinued and muscle relaxation antagonized with neostigmine and atropine.

Postoperative analgesia was provided by oral paracetamol 1 g every 8 hours and intravenous meperidine 0.3 to 0.4 mg/kg upon patient request or when verbal response pain scores exceeded 3 (on a 5-point scale with 0 = no pain and 5 = worst pain possible). Nonsteroidal agents (nonsteroidal anti-inflammatory drugs) were not given. Metoclopramide,10 mg intravenously, was the initial treatment for postoperative nausea and vomiting; 4 mg ondansetron was added if necessary.

### Measurements

Routine anesthetic monitoring was used as recommended by the American Society of Anesthesiologists.

Venous blood (7 ml) was sampled immediately after inserting the first peripheral cannula (T1), after intubation but before incision (T2), 2 hours after emergence (T3) and 24 hours (T4) after anesthesia. Samples were collected and centrifuged at 2,500×*g* for 10 minutes at room temperature; the resulting 3 to 4 ml plasma samples were stored at less than −20°C until assayed.

Plasma concentrations of interleukins and the CAMs soluble ICAM-1 and soluble VCAM-1 were measured by an ELISA technique using commercially available kits (Quantikine; R&D Systems, Minneapolis, MN, USA) as per the manufacturer’s instructions. Laboratory staff were unaware of study groups and were not involved in anesthetic management.

Detection limits for interleukins as given by the manufacturer were as follows: IL-1, typically <1 pg/ml; IL-6,<0.7 pg/ml; IL-8,<1.5 pg/ml; IL-10,<3.9 pg/ml; and IL-13,<32 pg/ml. Intra-assay and inter-assay coefficients of variation were both <10%. The detection limits for soluble VCAM-1 ranged from 0.2 to 1.3 (mean =0.6) ng/ml and the limits for soluble ICAM-1 ranged from 0.05 to 0.25 (mean = 0.10) ng/ml, with intra-assay and inter-assay variation coefficients both <8% (Quantikine; R&D Systems).

Normal plasma concentrations of interleukins, as given by the manufacturer, are: IL-1β,1 to 4 pg/ml; IL-6, 0.7 to12 pg/ml; IL-8,4to 31 pg/ml; IL-13,32 to 62 pg/ml; and IL-10,4 to 8 pg/ml. Normal plasma concentrations of CAMs, as given by the manufacturer, are 349 to 991 ng/ml for soluble VCAM-1 and 99 to 320 ng/ml for soluble ICAM-1.

### Statistical analysis

Sample size was estimated from a pilot study with 22 patients per group. Calculated area-under-the-curve (AUC) values for IL-6 showed a 46 pg·hour/ml difference between the two groups (standard deviation = 60 and 62, respectively). Using a two-tailed α error of 0.01, we estimated that 41 patients per group would provide an 80% power. Anticipating some inadequate samples, we enrolled 44 patients in each study group*.*

Chi-square tests were used to assess correlations between dichotomous data. The distribution for continuous data was evaluated using the Kolmogorov–Smirnov test, with subsequent comparison by *t* test or Mann–Whitney *U* test as appropriate. AUC values over 24 hours and values at each measurement time were determined in individual patients using the preoperative concentration as the baseline; between-group AUC differences were subsequently determined using Mann–Whitney U tests.

Data are expressed as either mean ± standard deviation or median (95% confidence interval (CI)). *Post-hoc* comparisons with baseline values of biomarker concentrations recorded at two different postoperative time intervals were performed using the Wilcoxon test. A Bonferroni correction was used with a consequent reduction of the α threshold to 0.01.

Data analyses were performed using SPSS 17.0 (SPSS Inc., Chicago, IL, USA) and Medcalc 8.3.1.1 (MedCalc Software, Mariakerke, Belgium) statistical packages.

## Results

Ninety-one patients were enrolled in the study and were randomized (46 to isoflurane and 45 to TIVA). Three patients were excluded from analysis because they were discharged within 24 hours or acute inflammation of the gallbladder was identified intraoperatively; we thus present data from the remaining 88 patients (44 per group) who completed the study. Demographic data were similar in each anesthetic group, as were anesthetic and surgical management (Table 1).

**Table 1:** Demographic data of the study groups

	**Total intravenous anesthesia (*n *** **= 44)**	**Isoflurane (*n *** **= 44)**	***P *** **value**
Age (years)	52 ±12	46 ±14	0.19
Weight (kg)	74 ± 16	75 ± 16	0.72
Gender (female/male)	34/10	38/6	0.40
American Society of Anesthesiologists I/II	20/24	25/19	0.39
Anesthesia time (minutes)	54 ± 15	54 ± 14	0.90
Intraoperative Bispectral Index	43 ± 4	44 ± 4	0.22
Intraoperative mean arterial pressure (mmHg)	84 ± 17	78 ± 16	0.07
Intraoperative heart rate (beats/minute)	76 ± 14	74 ± 14	0.44
Intraoperative core temperature (°C)	36.5 ± 0.3	36.6 ± 0.3	0.13
Intraoperative intravenous fluids (l)	1.2 ± 0.2	1.3 ± 0.2	0.47
Intraoperative remifentanil (μg/kg/minute)	0.3 ± 0.06	0.3 ± 0.05	1.00

Data expressed as mean ± standard deviation or number of patients.

Propofol targeted plasma concentrations in the target-controlled infusion TIVA group averaged 2.4 ± 0.5 μg/ml. The mean end-tidal isoflurane level in patients assigned to volatile anesthesia was 1.1 ± 0.3%.

None of the patients became hypothermic during operation. The mean intraoperative temperature was 36.6 ± 0.2°C.

Pre-induction plasma cytokine and adhesion molecule concentrations were similar in the two study groups (Table 2).

**Table 2:** Pre-induction interleukin and cell adhesion molecules plasma concentrations

	**Total intravenous anesthesia (*n *** **= 44)**	**Isoflurane (*n *** **= 44)**
Il-1β (pg/ml)	1.0 (1.02 to 1.18)	1.0 (0.99 to 1.31)
IL-6 (pg/ml)	3.4 ( 2.6 to 5.5)	2.2 (1.2 to 2.7)
IL-8 (pg/ml)	19.3 (12.4 to 34.6)	16.7 (12.1 to 24.0)
IL-10 (pg/ml)	6.1 (5.1 to 6.9)	4.8 (4.1 to 6.0)
IL-13 (pg/ml)	30.6 (27.2 to 34.0)	33.7 (28.8 to 35.8)
Soluble ICAM-1 (ng/ml)	69.0 (65.2 to 76.6)	66.1 (60.74 to 74.7)
Soluble VCAM-1 (ng/ml)	383 (349.03 to 408)	362 (335 to 402)

Data expressed as median (95% confidence interval). ICAM, intracellular adhesion molecule; VCAM, vascular cell adhesion molecule.

**Figure 1:**
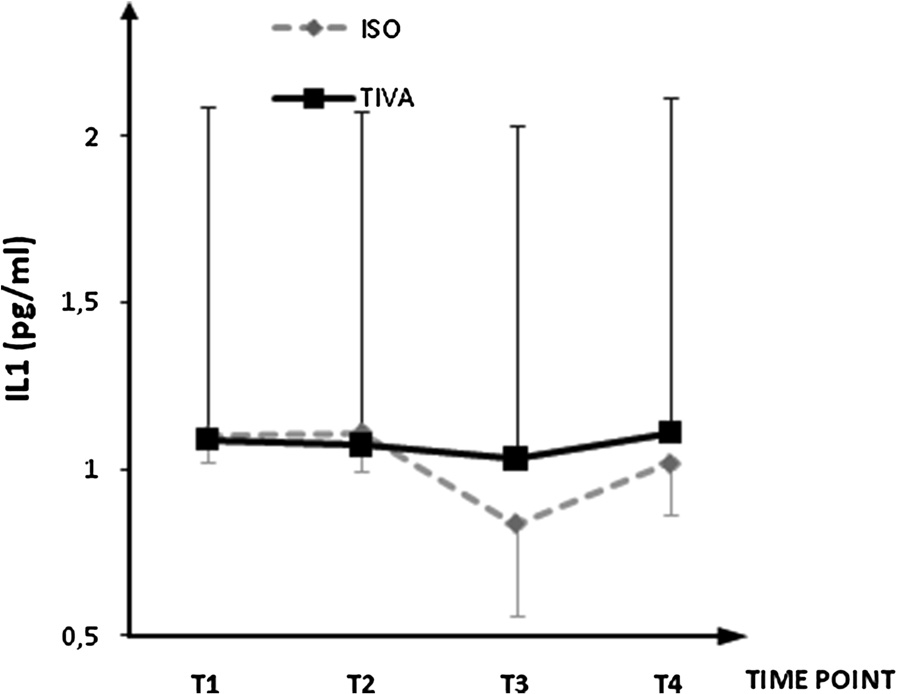
**Plasma IL-1β concentrations.** Data expressed as median (95% confidence interval). T1 = before induction; T2 = immediately after induction; T3 = at 2 hours after skin closure; T4 = at 24 hours after skin closure. Area under the curve: isoflurane (ISO) = 3.3, total intravenous anesthesia (TIVA) = 3.4.*P*= 0.428.

**Figure 2:**
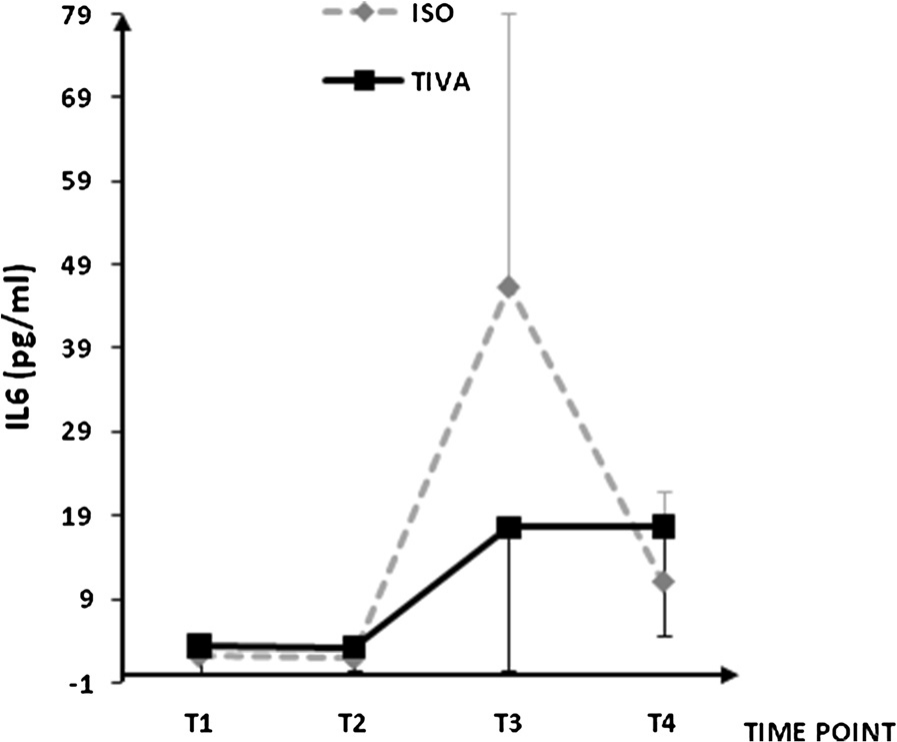
**Plasma IL-6 concentrations.** Data expressed as median (95% confidence interval).T1 = before induction; T2 = immediately after induction; T3 = at 2 hours after skin closure; T4 = at 24 hours after skin closure. Area-under-the-curve: isoflurane (ISO) = 78, total intravenous anesthesia (TIVA)= 33. *P* = 0.006.

**Figure 3:**
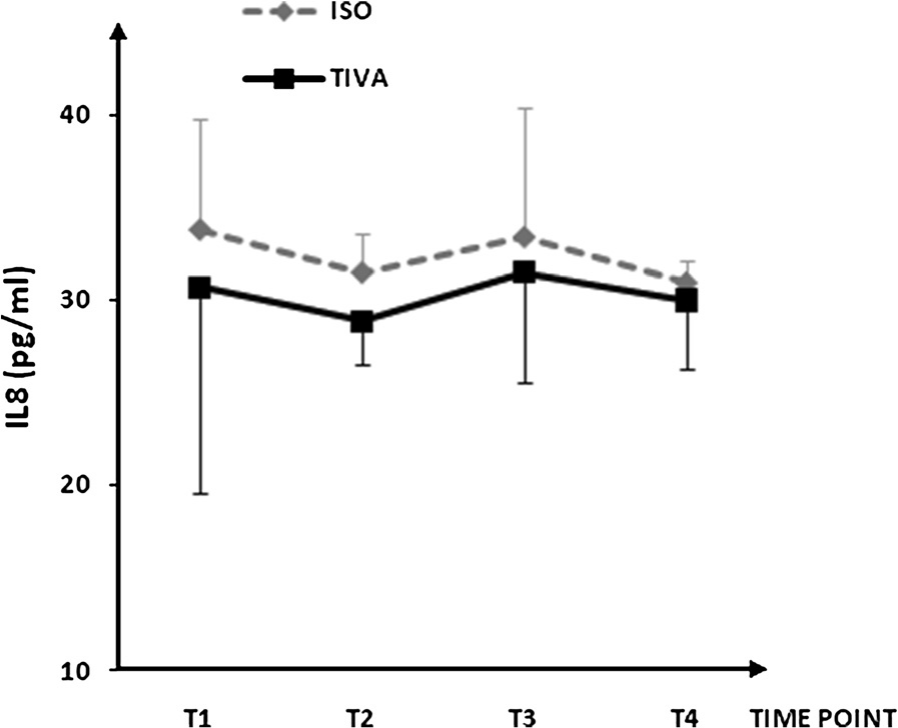
**Plasma IL-8 concentrations.** Data expressed as median (95% confidence interval). T1 = before induction; T2 = immediately after induction; T3 = at 2 hours after skin closure; T4 = at 24 hours after skin closure. Area-under-the- curve: isoflurane (ISO) = 54, total intravenous anesthesia (TIVA) = 49. *P* = 0.285.

**Figure 4:**
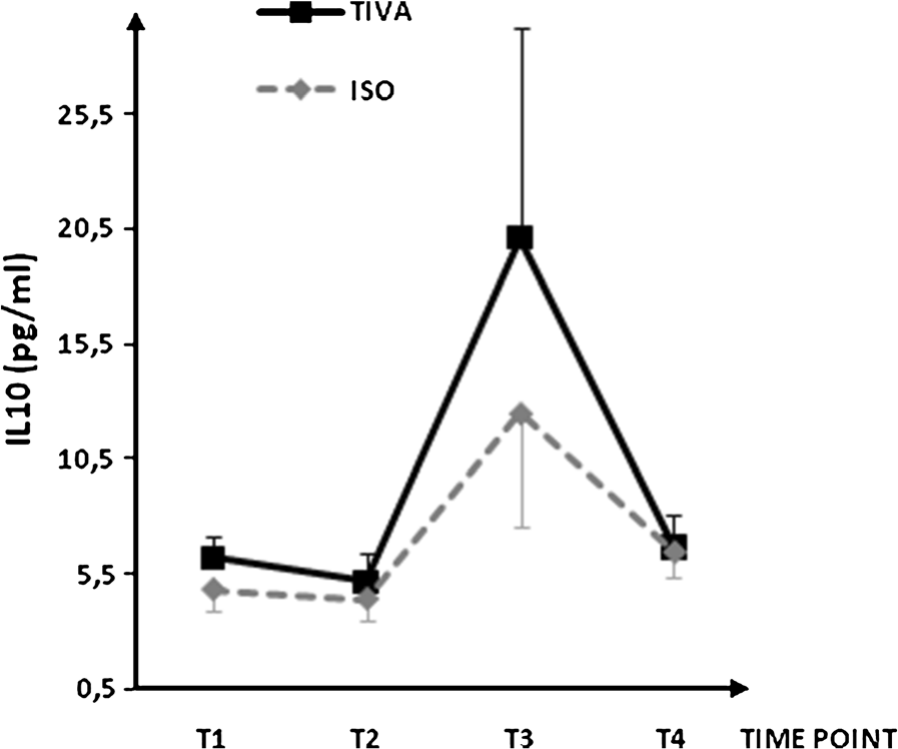
**Plasma IL-10 concentrations.** Data expressed as median (95% confidence interval). T1 = before induction; T2 = immediately after induction; T3 = at 2 hours after skin closure; T4 = at 24 hours after skin closure. Area-under-the-curve: isoflurane (ISO) = 26, total intravenous anesthesia (TIVA) = 37. *P* = 0.151.

**Figure 5:**
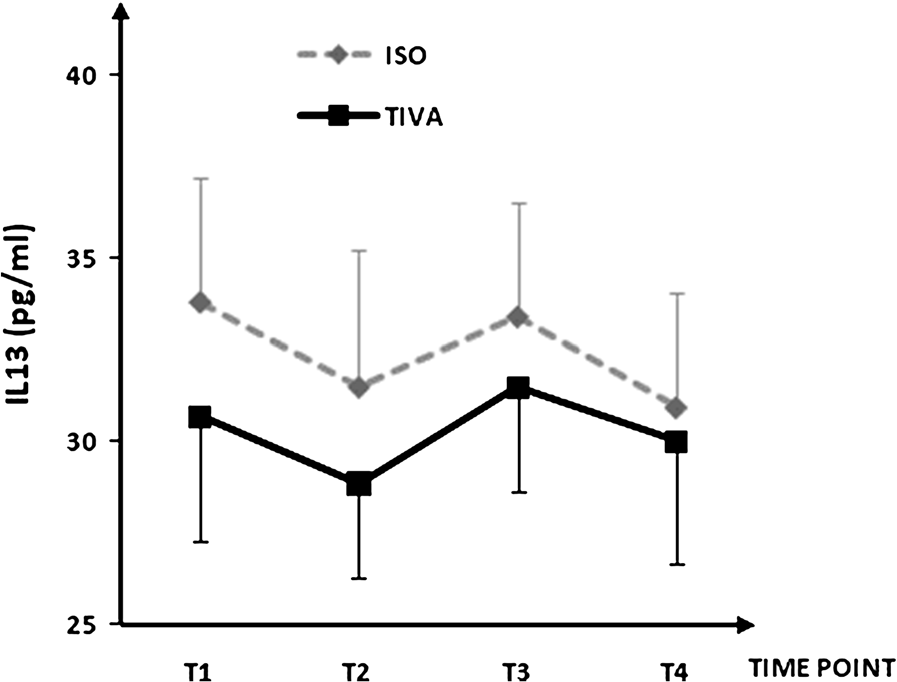
**Plasma IL-13 concentrations.** Data expressed as median (95% confidence interval). T1 = before induction; T2 = immediately after induction; T3 = at 2 hours after skin closure; T4 = at 24 hours after skin closure. Area-underthe- curve: isoflurane (ISO) = 101, total intravenous anesthesia (TIVA) = 93. *P* = 0.218.

**Figure 6:**
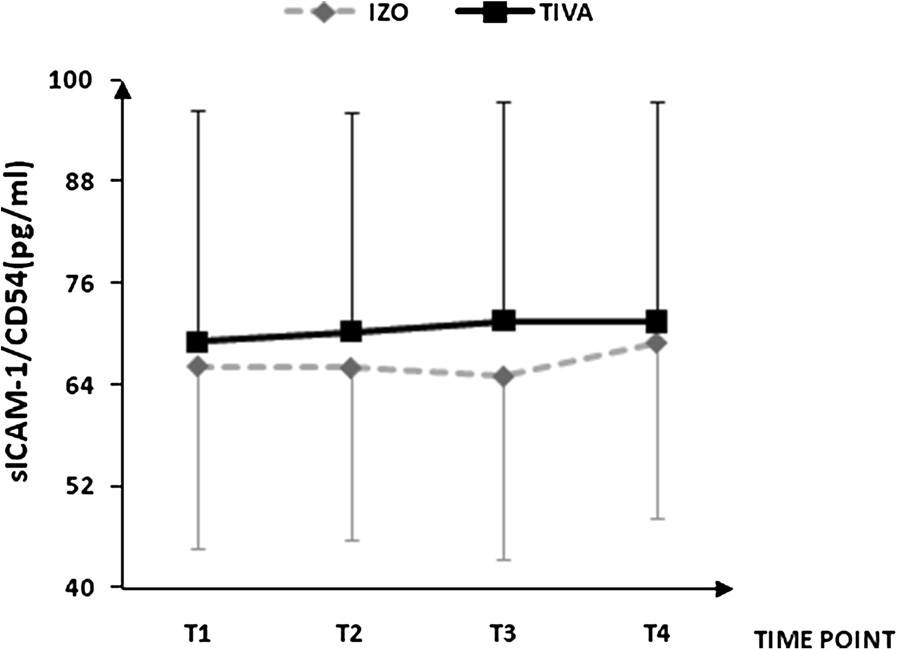
**Plasma soluble intercellular adhesion molecule-1 concentrations.** Data expressed as median (95% confidence interval). T1 = before induction; T2 = immediately after induction; T3 = at 2 hours after skin closure; T4 = at 24 hours after skin closure. Area-under-the-curve: isoflurane (ISO)= 197, total intravenous anesthesia (TIVA)= 213. *P* = 0.488. ICAM, intracellular adhesion molecule.

**Figure 7:**
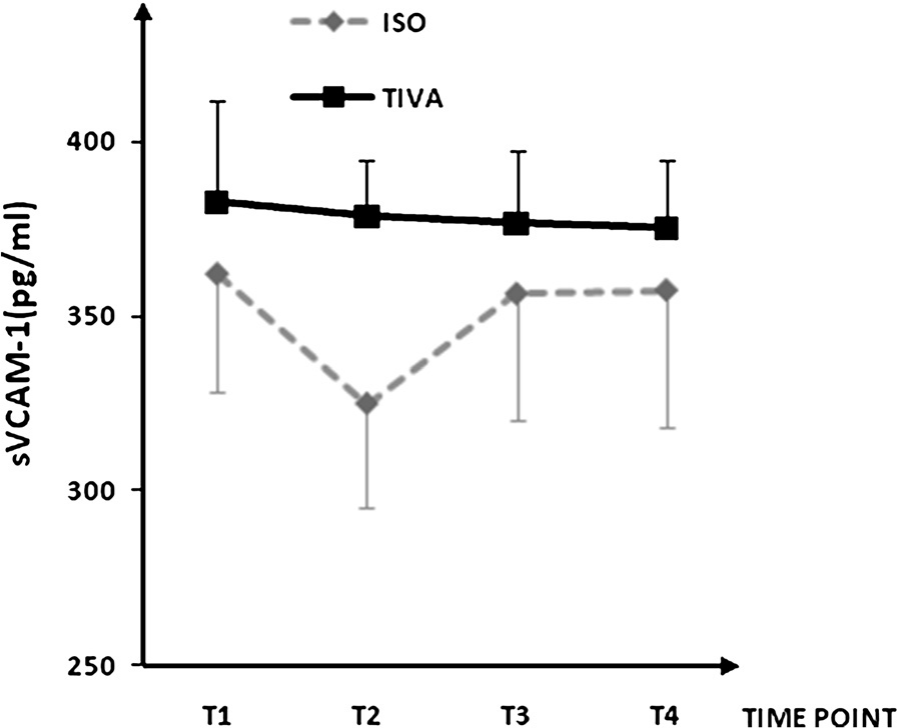
**Plasma solublevascular cell adhesion molecule-1 concentrations.** Data expressed as median (95% confidence interval). T1 = before induction; T2 = immediately after induction; T3 = at 2 hours after skin closure; T4 = at 24 hours after skin closure. Area-under –the-curve: isoflurane (ISO) = 1029, total intravenous anesthesia (TIVA) = 1139. *P* = 0.226. VCAM, vascular cell adhesion molecule.

Plasma concentrations for IL-1β, IL-6, IL-8, IL-10, IL-13, soluble ICAM-1, and soluble VCAM-1are shown in Figures 1, 2, 3, 4, 5, 6, and 7, respectively. The largest cytokine responses to surgery were observed for IL-6 and IL-10, each with significant peak concentrations 2 hours after surgery. IL-6 was significantly greater in patients assigned to isoflurane: 46.4 (95% CI: 34.3 to 70.7) pg/ml versus 17.6 (95% CI: 11.9 to 20.7) pg/ml (*P*<0.01). In contrast IL-10 was greater in patients assigned to TIVA: 20.1 (95% CI:14.2 to 32.4) pg/ml versus 12.4 (95%CI:8.8 to 18.7) pg/ml (*P* = 0.03). By 24 hours after surgery, concentrations were similar in each group and similar to baseline values, except for IL-6 that remained significantly increased in both groups (*P*<0.001) and for IL-10 that remained significantly increased in patients given isoflurane (*P*= 0.009). IL-4 concentrations were below the detection limits of our assay at all times and are therefore not reported.

Between-group comparisons of the cytokine plasma concentration AUC for every measured parameter in the study groups are showed in Table 3; the only significant difference was for IL-6 (*P* = 0.006), with values being greater in patients assigned to isoflurane anesthesia.

**Table 3:** Area under the curve between-group comparisons

	**Total intravenous anesthesia (*n *** **= 44)**	**Isoflurane (*n *** **= 44)**	***P*** **value**
IL-1β (pg·hour/ml)	3.4 (3.0 to 4.3)	3.3 (2.9 to 4.0)	0.428
IL-6 (pg·hour/ml)	33.0 (21.6 to 44.9)	78.0 (52.2 to 109.1)	**0.006**
IL-8 (pg·hour/ml)	49.0 (39.6 to 67.0)	54.0(47.2 to 82.6)	0.285
IL-10 (pg·hour/ml)	37.0 (23.8 to 55.4)	26.0 (21.6 to 42.1)	0.151
IL-13 (pg·hour/ml)	93.0 (83.4 to 101.3)	101.0 (89.8 to 112.1)	0.218
Soluble ICAM-1(ng·hour/ml)	213 (188.0 to 242.3)	197 (178.9 to 220.9)	0.488
Soluble VCAM-1(ng·hour/ml)	1,139 (1,029 to 1,188)	1,029 (947 to 1,163)	0.226

Data expressed as median (95% confidence interval). Area-under-the-curve based on preoperative values. Mann–Whitney U tests were used for between-group comparisons; because multiple comparisons were made, *P*<0.01 was considered statistically significant. Bold data are significant.

## Discussion

The possibility that anesthetic management influences long-term outcomes in surgical patients is intriguing, but remains largely speculative. Nonetheless, there is limited (and often controversial) evidence – or at least plausible mechanisms – to suggest that anesthetic management might influence diverse outcomes including wound infection [30-32], major cardiac complications and strokes [33], brain development [34], cancer recurrence [35-37], and mortality [30,38]. Because the inflammatory response to surgery seems likely to be an important potential mechanism, and possibly even a common pathway for many outcomes, we evaluated the cytokine responses in patients randomly assigned to TIVA or volatile anesthesia.

Based on our primary AUC analysis, the only cytokine that differed significantly as a function of anesthetic approach was IL-6, and the increase was only marginally statistically significant (*P*= 0.006 with an α threshold of 0.01 because of multiple comparisons). Furthermore, the factor of two increase – which is small by the standards of cytokines – seems unlikely to be clinically important. Judging by cytokine responses, our results thus suggest that the choice of TIVA versus volatile anesthesia only slightly alters the inflammatory response to surgery.

Within-group comparisons showed that IL-6 increased significantly at 2 hours postoperatively in both groups, but that the increase was significantly greater in patients assigned to isoflurane anesthesia. IL-10 was also significantly increased in both groups after 2 postoperative hours, but with greater plasma concentrations in patients given TIVA [3]. At 24 hours postoperatively, IL-6 remained increased in both study groups while IL-10 remained increased only in the inhalation group, probably to counteract the increase in IL-6; however, the increases were marginal.

Our results are generally consistent with previous reports by Ke and colleagues, Gililand and colleagues, and Crozier and colleagues [20-22]. For example, Ke and colleagues reported similar responses for IL-6 and IL-10 during laparoscopic cholecystectomy. Results reported by Gililand and colleagues in abdominal surgery and by Crozier and colleagues after abdominal hysterectomy were also generally similar. In contrast, Helmy and colleagues reported that IL-6 does not increase after laparoscopic cholecystectomy [39]. Potential explanations include use of a different kit for interleukin assays and variations in surgical technique.

Although there were differences in anesthetic protocols and type of surgery, Deegan and coworkers observed IL-10, IL-8, and IL-13 responses similar to ours when propofol and paravertebral anesthesia was compared with volatile anesthesia [35]. However, they also found that IL-6 concentrations did not much differ as a function of anesthetic dose. Potential explanations include their use of regional anesthesia rather than TIVA, breast surgery rather than abdominal surgery, and the fact that all their patients had cancer, which *per se* can depress immune responses. Furthermore, in our study neither soluble ICAM-1 nor soluble VCAM-1 differed significantly as an effect of anesthetic technique.

A possible explanation for the results on interleukins may consist of the antioxidants and anti-inflammatory effects of propofol [24,40] as compared with immune effects of inhalation agents [41]. As for adhesion molecules, our results with inhalation anesthesia are similar to other studies [42], confirming that isoflurane has an inhibitory effect on CAMs. Moreover, we have demonstrated that there are no differences between inhalation anesthesia and TIVA. The anti-inflammatory effects of propofol may thus involve mechanisms other than adhesion molecules.

Directly comparing our results with previous publications is difficult since each study evaluated different anesthetic protocols, different surgical interventions, and used different cytokine assays. Nonetheless, our results are thus generally consistent with the previous literature and are among the largest that evaluated a single typical operation.

Our study does have some limitations. A low dose of dexamethasone (4 mg) was given to all patients for prophylaxis against postoperative nausea and vomiting, as is common in clinical practice. It is wellknown that steroids are immunosuppressive and may have ameliorated the inflammatory response to surgery. Observed differences between the randomized TIVA and volatile anesthetic groups remain valid, but it remains possible that responses in both groups would be more impressive in patients not given steroids. A more serious consideration is that laparoscopic cholecystectomies produce only a moderate amount of tissue injury, and cholecystectomies presumably provoke a smaller inflammatory response than larger operations. They nonetheless well represent the types of surgery that are most commonly performed. On the contrary, having only a minor inflammatory response due to surgery, the differences may be more attributable to anesthetic technique.

Immune response is a mosaic in which interleukins and adhesion molecules are but one piece. However, it is an important piece because exaggerated or abnormally low cytokine concentrations may have a substantial effect on patient outcome. For example abnormally increased levels of IL-6 are involved in systemic inflammatory response with impact on outcome and postoperative complications, and even on prognosis and mortality in cancer patients [43,44]. However, we observed relatively small differences between isoflurane anesthesia and TIVA, and only over a short period of time; whether this difference is clinically important remains unknown — but seems somewhat unlikely.

In summary, IL-6 and IL-10 increased significantly 2 hours after incision. There were no other statistically significant or clinically important perioperative increases. The only significant difference in cytokine concentrations related to anesthetic management was a greater increase in the IL-6 AUC with isoflurane anesthesia than with TIVA. However, the increase was only a factor-of-two, which is small by cytokine standards. The AUC concentrations were greater for IL-10 (*P*= 0.15), soluble ICAM-1 (*P* = 0.49), and soluble VCAM-1 (*P* = 0.23) in patients assigned to TIVA, although not significantly.

## Conclusion

TIVA significantly reduced the increase in IL-6 during the perioperative period as compared with isoflurane. IL-10 and the adhesion molecules were increased, although not significantly. This effect may be favorable in some patients (for example, increased systemic inflammatory response). Further studies on larger groups of patients and for more extensive surgical interventions are needed for a better evaluation of the extent of this effect and its clinical impact.

## Abbreviations

AUC: Area under the curve; CAM: Cell adhesion molecule; CI: Confidence interval; ELISA: Enzyme-linked immunosorbent assay; IL: Interleukin; ICAM: Intercellular adhesion molecule; NK: Natural killer; TIVA: Total intravenous anesthesia; VCAM: Vascular cell adhesion molecule.

## Competing interests

The authors declare that they have no competing interests.

## Authors’ contributions

DCI conceived the study, design study protocol, and acquisition of data, and drafted the manuscript. SCDM contributed to acquisition of data by enrolling patients and to drafting the manuscript. ANIH contributed to acquisition of data (sample preservation, storage) and the database. TNM contributed to the database and statistical analysis and interpretation of the data. NAM was responsible for immunological analysis of blood samples and interpretation. DIS revised critically the manuscript for important intellectual content and drafted parts of it. All authors read and approved the final manuscript.
